# A step change in the transfer of interspecific variation into wheat from *Amblyopyrum muticum*

**DOI:** 10.1111/pbi.12606

**Published:** 2016-08-25

**Authors:** Julie King, Surbhi Grewal, Cai‐yun Yang, Stella Hubbart, Duncan Scholefield, Stephen Ashling, Keith J. Edwards, Alexandra M. Allen, Amanda Burridge, Claire Bloor, Alessandro Davassi, Glacy J. da Silva, Ken Chalmers, Ian P. King

**Affiliations:** ^1^ Division of Plant and Crop SciencesSchool of BiosciencesThe University of Nottingham, Sutton Bonington CampusLoughboroughUK; ^2^ Life SciencesUniversity of BristolBristolUK; ^3^ Affymetrix UK LtdHigh WycombeUK; ^4^ Federal University of PelotasPelotasBrazil; ^5^ School of Agriculture, Food and WineThe University of AdelaideAdelaideSAAustralia

**Keywords:** Wheat, Introgression, wild relatives, genotyping, synteny

## Abstract

Despite some notable successes, only a fraction of the genetic variation available in wild relatives has been utilized to produce superior wheat varieties. This is as a direct result of the lack of availability of suitable high‐throughput technologies to detect wheat/wild relative introgressions when they occur. Here, we report on the use of a new SNP array to detect wheat/wild relative introgressions in backcross progenies derived from interspecific hexaploid wheat/*Ambylopyrum muticum* F_1_ hybrids. The array enabled the detection and characterization of 218 genomewide wheat/*Am. muticum* introgressions, that is a significant step change in the generation and detection of introgressions compared to previous work in the field. Furthermore, the frequency of introgressions detected was sufficiently high to enable the construction of seven linkage groups of the *Am. muticum* genome, thus enabling the syntenic relationship between the wild relative and hexaploid wheat to be determined. The importance of the genetic variation from *Am. muticum* introduced into wheat for the development of superior varieties is discussed.

## Introduction

Hexaploid bread wheat, which is an allopolyploid composed of three distinct genomes, that is the AA genome from *Triticum urartu*, the BB genome from an *Aegilops speltoides*‐like progenitor and the DD genome from *Aegilops tauschii* (Dvorak and Zhang, 1990; Dvorak *et al*., 1993; McFadden and Sears, 1946), evolved only once or at best a few times approximately 10 000 years ago (Charmet, 2011). As a result, wheat has been through a severe genetic bottleneck with the sum total of genetic variation present in the species today being a direct result of only 10 000 years of genetic mutation and through possible outcrossing events that may have occurred with other species, for example tetraploid wheat. In addition, the gene pool of modern cultivated wheat has been further restricted through selection for specific agronomically important traits, for example free threshing (Charmet, 2011; Cox, 1997).

Wheat is one of the world's leading sources of food, and thus, the narrow gene pool available for the development of superior varieties is of major concern heightened by increasing global population predictions. In the past, breeders have had considerable success in producing higher yielding varieties with the limited variation available. However, there is growing evidence that wheat yields are plateauing and that this is a direct result of the exhaustion of the available genetic variation compounded by environmental change (Brisson *et al*., 2010; Charmet, 2011; Ray *et al*., 2013). Thus, there is an urgent need to identify new sources of genetic variation that can be used to develop superior wheat varieties.

Wheat is related to a large number of other species many of which are wild and uncultivated. These wild relatives, unlike wheat, provide a vast and untapped reservoir of genetic variation for potentially most, if not all, agronomically important traits (Friebe *et al*., 1996; Jauhar and Chibbar, 1999; Qi *et al*., 2007; Schneider *et al*., 2008). In the past, attempts have been made to exploit the genetic variation from these wild species.

Normally, recombination in wheat is restricted to identical homologous chromosomes from the same genome due to the presence of the *Ph1* locus located on the long arm of chromosome 5B of wheat (Al‐Kaff *et al*., 2008; Griffiths *et al*., 2006; Riley and Chapman, 1958; Sears, 1976; Sears and Okamoto, 1958). Thus, the *Ph1* locus normally has to be removed before homoeologous recombination between the chromosomes of a wild relative and wheat can occur (Al‐Kaff *et al*., 2008; Sears, 1977). However, some species such as *Amblyopyrum muticum* [(Boiss.) Eig. (*Aegilops mutica* Boiss.) (2*n* = 2*x* = 14; genome TT)] carry a gene(s) which supresses the *Ph1* locus, thus enabling recombination to occur directly between homoeologous chromosomes in interspecific *Am. muticum*/wheat F_1_ hybrids (Dover and Riley, 1972). Despite the ability to suppress the *Ph1* locus in wheat, very little genetic or trait analysis has been undertaken to date on *Am. muticum* with the exception of some addition and substitution lines (Iefimenko *et al*., 2013) and potential resistance against environmental stresses (Iefimenko *et al*., 2015) and fungal diseases such as powdery mildew (Eser, 1998).

Although there have been some notable successes (Garcia‐Olmedo *et al*., 1977 and Sears, 1955, 1972), only a fraction of the work has led to the development of new wheat varieties. The major blocks to the successful large‐scale genomewide exploitation of genetic variation from wild relatives have been firstly the lack of high‐throughput screening technology to quickly identify and characterize introgressions when they occur and secondly the apparent low frequency of recombination between the chromosomes of wheat and many of its wild relatives.

In the past, the identification of introgressions has relied on low‐throughput analyses with limited success. However, the development of next‐generation sequencing technologies and single nucleotide polymorphism (SNP) markers provides a mechanism for the detection of introgressions into wheat from its wild relatives as was demonstrated for chromosome 5 of *Aegilops geniculata* (Tiwari *et al*., 2014, 2015). The aim of the research described here was to use a whole‐genome introgression approach (e.g. King *et al*., 2013), that is attempt to transfer chromosome segments from the entire genome of *Am. muticum* into hexaploid wheat irrespective of any traits that the wild relative might carry and then to attempt to detect and characterize the introgressions via a new wheat/wild relative SNP array. This array was constructed using a subset of the SNPs described by Winfield *et al*. (2015) from their ultra‐high‐density Axiom^®^ genotyping array.

## Results

### Generation of introgressions

**Figure 1:**
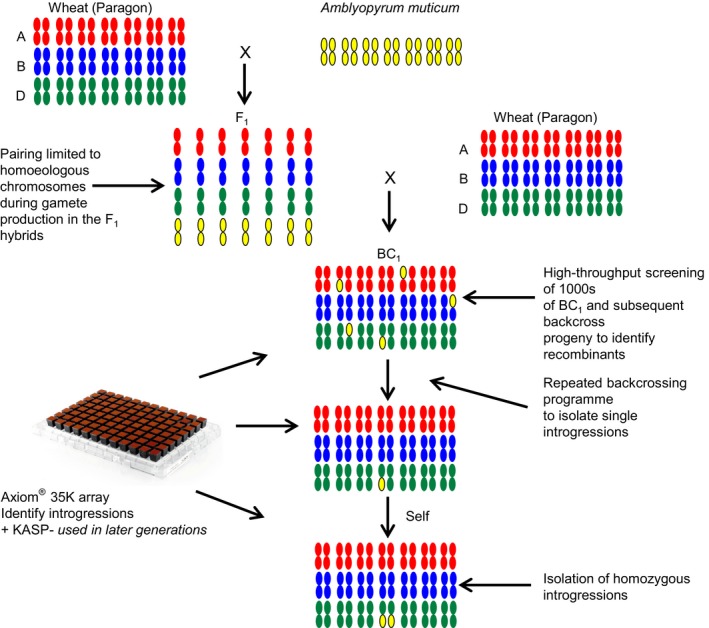
Wheat/wild relative introgression strategy.

In total, 1039 crosses (crossed ears) were made (Figure 1) resulting in the production of 8146 seeds (not including self‐seed). The number of seeds germinated, plants crossed and seed set, etc. is summarized in Table S1. The F_1_ interspecific hybrids between hexaploid wheat cv Paragon and *Am. muticum* showed the lowest germination rate—28.6%, as compared to the BC_1_, BC_2_ and BC_3_ generations—52.9%, 77.6% and 60.6%, respectively. In addition, the F_1_ hybrids also exhibited the highest levels of infertility (to obtain a broad indication of fertility, the number of crossed ears that produced seed was recorded for each generation), that is only 16.2% of F_1_ crossed ears produced seed as compared with 79.9%, 88.1% and 98.3% from the crossed ears of the BC_1_, BC_2_ and BC_3_ generations. A further indication of the infertility of the F_1_ was shown by the fact that this generation set no self‐seed in contrast to the other generations. Of the 98 F_1_ seeds germinated, only 11 reached maturity and set seed when pollinated with Paragon (Table S1). Thus, the total generation of *Am. muticum*/wheat introgressions in this program was limited to these 11 individuals and their derivatives. Of the 34 BC_1_ seeds generated from the 11 sexually partially viable F_1_ plants, 16 survived to maturity and set seed when pollinated with Paragon. In total, 781 BC_2_ seeds were obtained from 123 crosses onto these 16 BC_1_ plants. One hundred and sixteen of these BC_2_ seeds were then selected at random and germinated and subsequent pollinations with Paragon yielded 4137 BC_3_ seed. One hundred and twenty‐seven of these BC_3_ seeds were again selected at random and crossed to Paragon to generate 2947 BC_4_ seeds (Table S1).

### Detection of introgressions

An Axiom HD Wheat‐Relative Genotyping Array (a full description of which is provided in the Experimental Procedures) composed of SNPs showing polymorphism between several wheat varieties and ten wheat wild relatives [25 487 of the SNPs were polymorphic between *Am. muticum* and Paragon (Table 1) with the highest number for linkage group 2 (17.2%) and the fewest for linkage group 6 (11.4%) but with a relatively even spread over all seven linkage groups] was used to screen genomic DNA prepared from 167 samples, which included nine parental lines and 158 backcross lines between wheat and *Am. muticum*. Genotype calls were generated, and the sample call rate ranged from 80% to 97% with an average of 93.3% for the 167 samples. The lowest call rate was obtained for *Am. muticum* with an average of 80.1%.

**Table 1:** Number of polymorphic SNPs between *Am. muticum* and hexaploid wheat in total on the Affymetrix 35 K array and used in the linkage map of *Am. muticum*

	Linkage group 1	Linkage group 2	Linkage group 3	Linkage group 4	Linkage group 5	Linkage group 6	Linkage group 7	Total
All calls (% of total)	3254 (12.8)	4395 (17.2)	3825 (15.0)	3199 (12.6)	4182 (16.4)	2895 (11.4)	3717 (14.7)	25 487
PHR calls (% of total)	80 (13.1)	88 (14.4)	73 (11.9)	74 (12.1)	134 (21.9)	68 (11.1)	96 (15.7)	613

The scores for the probes were classified into one of six categories according to the cluster pattern produced by the Affymetrix software. Only the first group, Poly High Resolution (PHR), was considered as being optimum quality SNPs for genetic mapping purposes (see Experimental Procedures).

**Figure 2:**
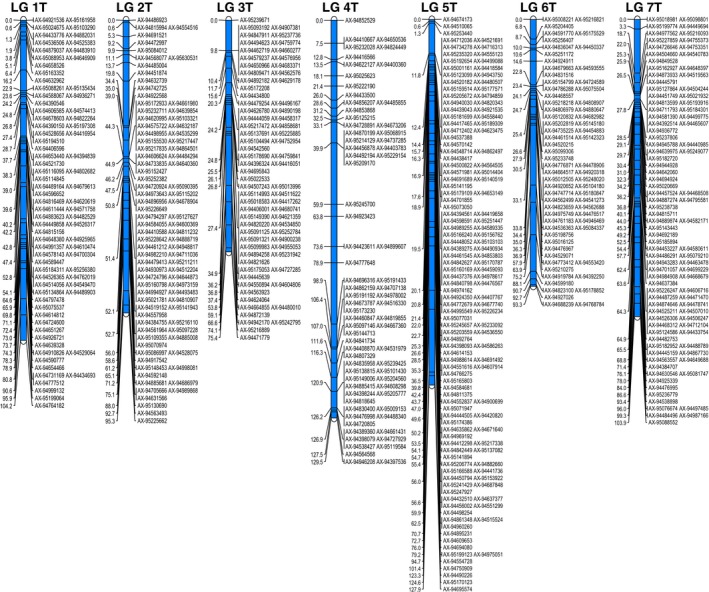
Genetic linkage map of *Am. muticum*. SNP marker names and cM distances for each of the seven linkage groups are also shown in supporting Table S2.

The PHR SNPs were used in map construction using JoinMap^®^ (van Ooijen, 2011) and resulted in seven linkage groups representing the seven *Am. muticum* chromosomes containing 613 SNP markers [Figure 2 (SNP marker names and cM distances for each of the seven linkage groups are also shown in Table S2)]. The cM lengths of linkage groups 1–7 were 104.2, 95.3, 75.4, 129.5, 127.9, 93.3 and 103.9, respectively. Hence, the total map length of this ‘frame’ was 729 cM with an average chromosome length of 104 cM. SNP markers were again well distributed over the seven linkage groups.

### Genomic *in situ* hybridization (GISH)

**Figure 3:**
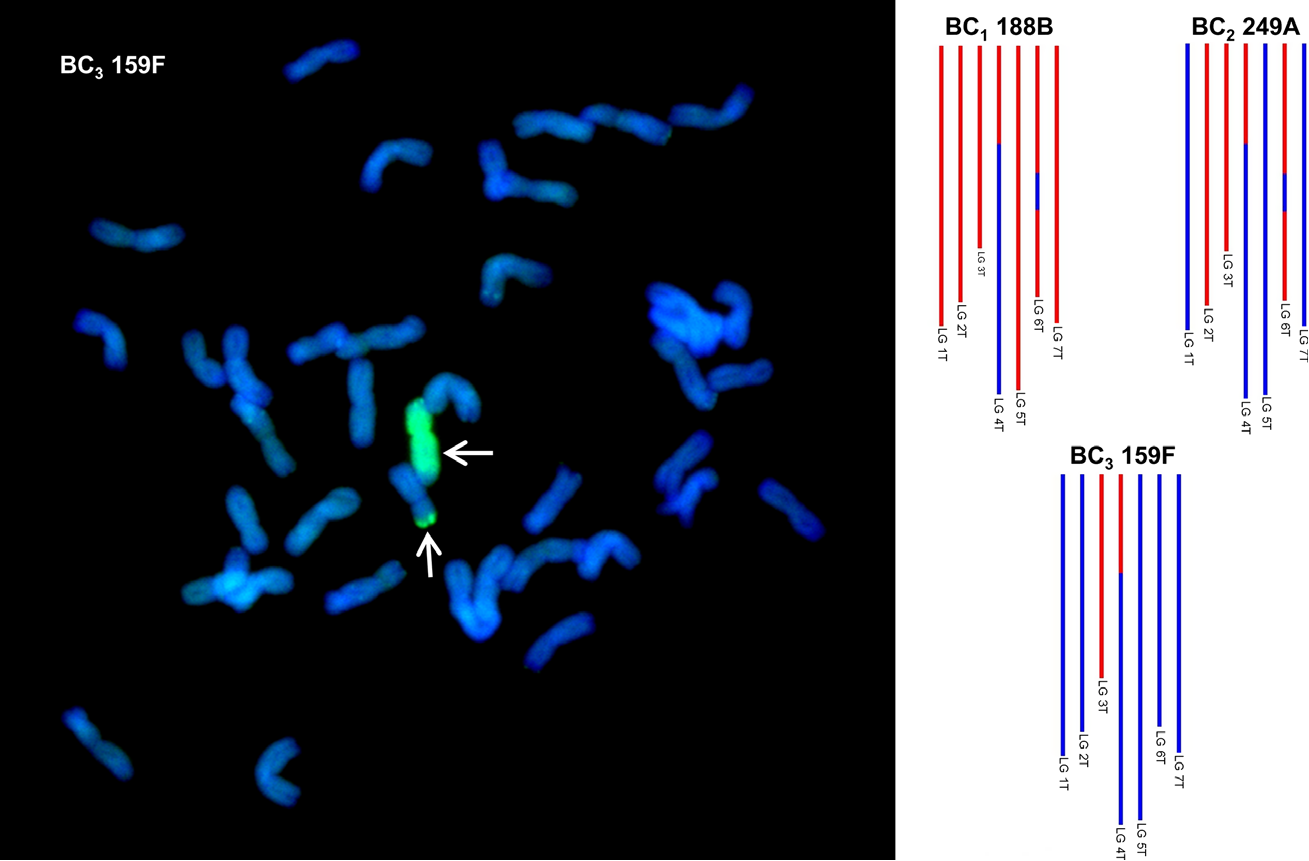
SNP characterization of *Am. muticum* introgressions in three consecutive generations, that is BC_1_, BC_2_ and BC_3_ and genomic *in situ* hybridisation image of the BC_3_ genotype. In the SNP characterization, red colour is used to represent the presence of an *Am. muticum* introgression, blue colour wheat. It should be noted that these diagrams cannot be used to assess which wheat chromosomes the *Am. muticum* segments have recombined with. The GISH image shows a metaphase spread of BC_3_ 159F probed with labelled genomic DNA of *Am. muticum*. Arrows show *Am. muticum* introgressions (green).

**Figure 4:**
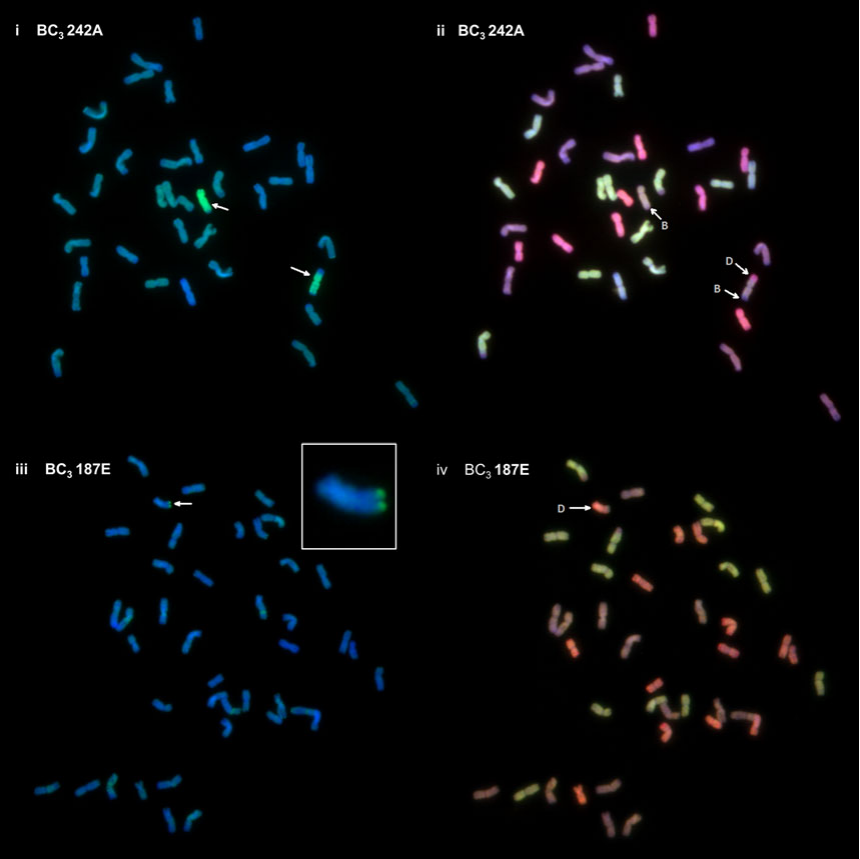
i–iv. Genomic *in situ* hybridization (GISH) showing recombination between *Am. muticum* and the B and D genomes of wheat. (i) GISH of complete one‐cell metaphase spread with labelled genomic *Am. muticum* as probe showing *Am. muticum* (green) introgressions (white arrows). (ii) Same metaphase spread as (i) with three‐colour GISH showing one *Am. muticum* introgression recombined with the B genome (purple) of wheat and the second introgression recombined with both the B (purple) and D (red) genomes of wheat. (iii) GISH of complete one‐cell metaphase spread with labelled *Am. muticum* as probe showing an *Am. muticum* (green) introgression (white arrow) [also shown in the magnified inset chromosome]. (iv) Same metaphase spread as (iii) with three‐colour GISH showing recombination between *Am. muticum* and the D (red) genome of wheat.

**Figure 5:**
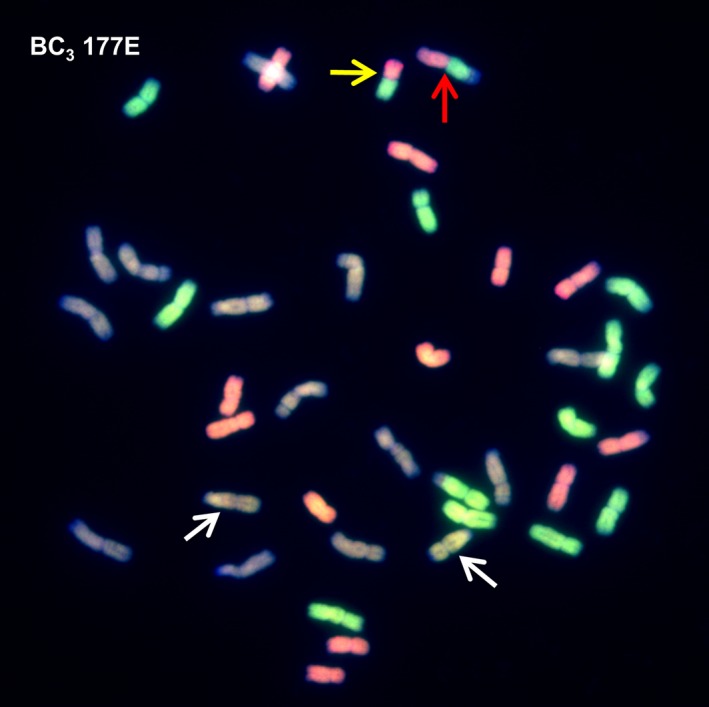
GISH image showing intergenomic recombination. The metaphase spread shows a 41 chromosome cell with 12 A genome chromosomes (green), 13 B genome chromosomes (purple) and 12 D genome chromosomes (red). There are also two *Am. muticum* introgressions (white arrows), one A/D recombinant chromosome (yellow arrow) and one B/A/D recombinant chromosome (red arrow).

To confirm the SNP analysis, genotyped BC_3_ individuals were selected and analysed by multicolour GISH. In each of the genotypes observed, the number of *Am. muticum* introgressions identified by SNP analysis corresponded exactly with the number of introgressions detected by GISH (Table 2, Figure 3). In all cases, the introgressions observed involved recombinant events between T genome chromosomes of *Am. muticum* and the B genome chromosomes of wheat (21 recombination events) or the D genome chromosomes of wheat (18 recombination events) (Figures 4i–iv). In four unrelated BC_3_ genotypes, one chromosome of *Am. muticum* was found to have recombined with both B and D genome chromosomes of wheat. No examples of recombinant events between the T genome chromosomes of *Am. muticum* and A genome chromosomes of wheat have to date been detected (of 22 genotypes containing introgressions). GISH also revealed the presence of intergenomic recombinant events between the A, B and D genomes of wheat (Figure 5). In the 29 BC_3_ genotypes analysed, four A/B translocations, ten A/D translocations and ten B/D translocations were observed.

**Table 2:** Number of introgressed segments from *Am. muticum* present in BC_3_ plants as detected by SNP genotyping and genomic *in situ* hybridization (GISH). The *Am. muticum* linkage group of each introgression is based on the SNP marker positions in wheat

Accession numbers of BC_3_ plants	Number of segments		*Am. muticum* linkagegroup of segment
	Genotyping	GISH	
157C	0	0	
157D	0	0	
157E	0	0	
159F	2	2	LG3, LG4
159G	1	1	LG4
159H	0	0	
163C	0	0	
163D	0	0	
163E	0	0	
172D	1	1	LG5
172E	3	3	LG1, LG3, LG5
177C	2	2	LG2, LG4
177D	1	1	LG4
177E	2	2	LG2, LG4
178C	1	1	LG7
178D	1	1	LG3
181C	1	1	LG6
182F	0	0	
182G	3	3	LG1, LG2, LG7
182H	1	1	LG2
187E	1	1	LG2
238A	2	2	LG6, LG7
238B	2	2	LG6, LG7
240A	3	3	LG4, LG5, LG7
241A	3	3	LG4, LG5, LG7
242A	2	2	LG5, LG7
243A	2	2	LG1, LG5
243B	3	3	LG1, LG5, LG6
243C	2	2	LG1, LG5
246	4	4	LG3, LG4, LG5, LG6

### Syntenic relationship between wheat and *Am. muticum*

**Figure 6:**
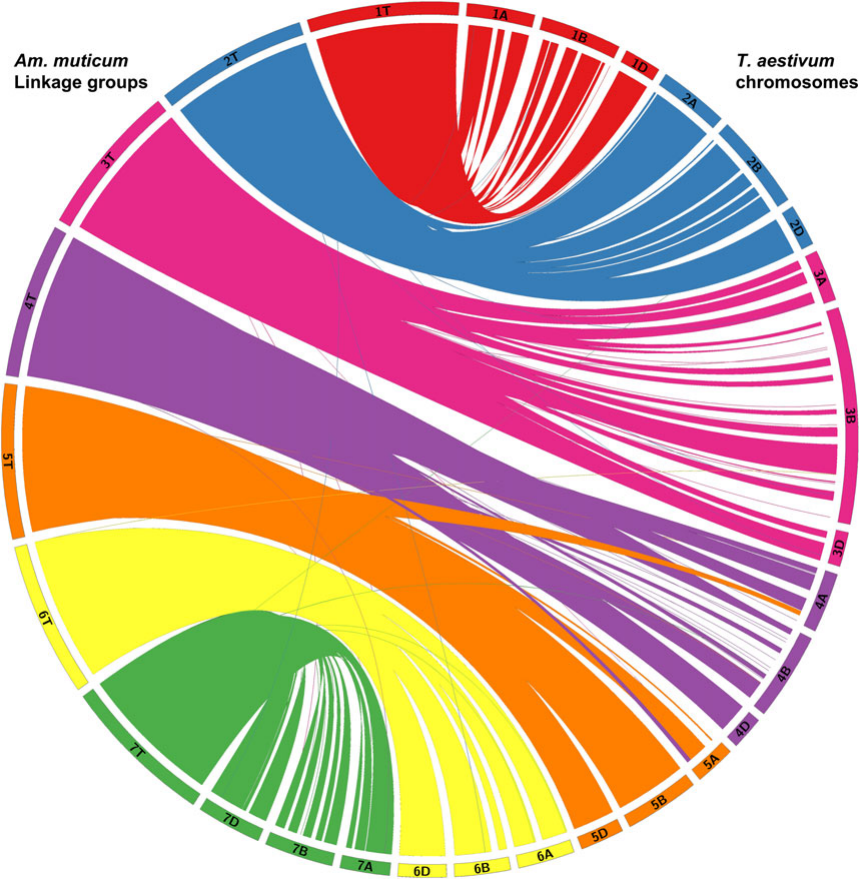
Synteny of *Am. muticum* (genetic position in cM) with hexaploid wheat (physical position in Mb) [visualized using Circos v. 0.67; Krzywinski *et al*., 2009].

Figure 6 shows the syntenic relationships between the seven linkage groups of *Am. muticum* and the seven linkage groups of each of the three genomes of wheat with large ‘ribbons’ showing significant synteny. Some gene rearrangements are indicated in the diagram where usually single markers cross map to noncollinear positions on the wheat chromosomes. The only major disruption to the syntenic relationship between these two species is that *Am. muticum* does not carry the 4/5/7 translocation observed for chromosomes 4A, 5A and 7B of wheat (Liu *et al*., 1992; Naranjo *et al*., 1987). Thus, this analysis represents a close syntenic relationship between *Am. muticum* and the A, B and D genomes of wheat.

## Discussion

Since the initial discovery of the *Ph1* chromosome pairing control locus in wheat (Riley and Chapman, 1958; Sears and Okamoto, 1958), many attempts have been made to unlock the genetic variation in wild relatives for wheat improvement. However, while introgression into wheat has been used successfully, albeit haphazardly in the past, for example leaf rust resistance transfer from *Aegilops umbellulata* (Sears, 1955, 1972), the potential of wild relatives has remained virtually untapped. The failure for the systematic exploitation of wild relatives has been the absence of appropriate high‐throughput technologies to screen for, and specifically identify, introgression events (King *et al*., 2016). The Affymetrix wheat/wild relative array used in this present study enabled the identification and characterization of genomewide introgressions of various sizes (from large to very small). The shotgun introgression approach described has resulted in the generation of a high level of wheat/*Am. muticum* recombinant chromosomes. The ability to detect these introgressions is a direct result of using the SNP array, that is large numbers of markers to detect genomewide introgressions have not been previously available.

In the work described, recombination between the wheat and *Am. muticum* chromosomes, leading to introgressions, was expected to occur in female gametes of wheat/*Am. muticum* F_1_ interspecific hybrids (rather than using individual wheat/wild relative addition or substitution lines that have commonly been used in the past (King *et al*., 2016). As these F_1_ hybrids lack homologous chromosome pairs, the only recombination that can occur is between chromosomes from different genomes, that is between the A, B, D genomes of wheat and the T genome of *Am. muticum*. As a result of the absence of homologous chromosomes, we hypothesized that this strategy might lead to an enhanced frequency of introgression. However, the drawback of this approach is that as the frequency of recombination between chromosomes from different genomes is likely to be very low, this would lead to significant infertility in the F1, that is low recombination would result in the failure of normal disjunction of chromosomes at anaphase I of meiosis leading to the production of unviable, unbalanced gametes.

**Figure 7:**
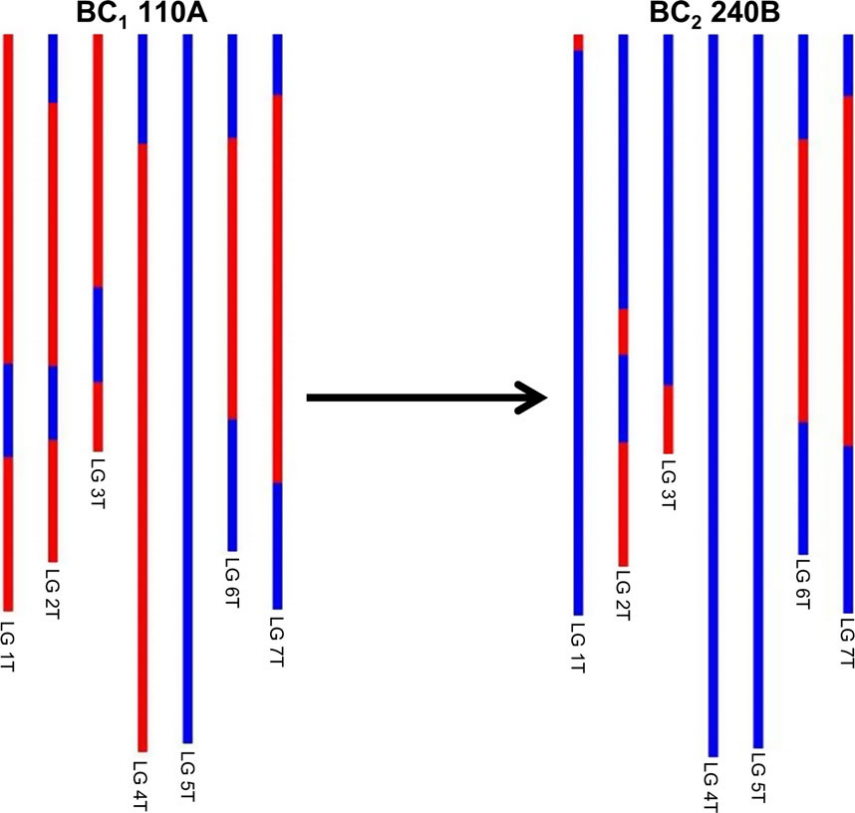
SNP analysis of *Am. muticum* introgressions in two consecutive generations (BC_1_ and BC_2_) showing recombination (involving linkage group 2) has occurred during gametogenesis in the BC_1_ genotype. Red colour shows the presence of *Am. muticum* introgressions, and blue colour shows wheat chromosomes.

The F_1_ individuals showed very low fertility as predicted, for example the frequency of seed set per crossed ear of the F_1_ hybrids was only 16.2% as compared to 79.67%, 88.09% and 98.25% for the BC_1_, BC_2_ and BC_3_ generations, respectively (Table S1). As a result of the low fertility of the F_1_ hybrids, only 34 BC_1_ individuals were generated, and of these, only 16 plants grew to maturity and set seed. Thus, the total number of introgressions that could be generated would be limited to the 16 female F_1_ gametes that gave rise to these 16 BC_1_ plants if no further recombination occurred in later generations, that is in the gametes of the BC_1_, BC_2_ and BC_3_ generations. However, unexpectedly, genetic mapping indicated that a very high frequency of interspecific recombination had occurred between the chromosomes of wheat and those of *Am. muticum*, that is it was possible to assemble seven linkages groups—something that has not been possible to achieve previously in this field of research and thus representing a step change in the generation, detection and characterization of wheat/wild relative introgressions. From the genetic mapping of the SNP markers, it was possible to estimate that we had generated 218 wheat/*Am. muticum* introgressions spanning the entire genome of the wild relative. The genetic map also allowed us to characterize and track the introgressions as can be seen in Figure 3. However, it should be noted that the germplasm used to generate these linkage maps did not constitute proper mapping families and in fact we combined different generations in order to have sufficient numbers. Therefore, the cM distances should be treated with considerable caution. Analysis of the SNP data revealed that the majority of introgressions generated were present in the 16 BC_1_ individuals, that is significant levels of recombination occurred in the gametes of the F_1_ hybrids. However, SNP analysis also indicated that further recombination between wheat/*Am. muticum* chromosomes was also continuing to occur in later generations (Figure 7).

In summary, the very high frequency of introgression detected via the SNP array offsets any problems in the low fertility observed in the F_1_ interspecific hybrids, and hence, this low fertility can be simply overcome by increasing the number of crosses made to the F_1_ individuals in an introgression programme.

SNP markers from the array are being converted into KASP markers, thus allowing us to track individual introgressions through the process of backcrossing and selfing as we are able to ‘tag’ the introgressions with selected markers. The advantage of this approach as opposed to the one used by Wendler *et al*. (2014, 2015) to detect *Hordeum bulbosum* introgressions in *H. vulgare*, that is that of applying genotyping by sequencing or exome capture re‐sequencing and mapping SNP variation to a reference genome, is that we are able to use the one dedicated array to identify and characterize introgressions from all ten of the wild relative species we are currently working with, without the need for a reference genome. To date, we have screened approximately 2500 individual genotypes via the array.

The presence of wheat/*Am. muticum* introgressions that were identified via SNP analysis and genetic mapping was confirmed by multicolour GISH (Figure 4i–iv). In addition, intergenomic recombinants between the A, B and D genomes of wheat were also observed (Figure 5). Thus, as part of the ongoing programme, we are backcrossing lines to obtain individuals with single introgressions but which have lost the A, B and D intergenomic recombinants. SNP analysis revealed a close syntenic relationship between all three genomes of wheat and the T genome of *Am. muticum* with the exception that the latter does not carry the 4A/5A/7B translocation seen in wheat (Figure 6). However, to date, GISH has only identified *Am. muticum* introgressions between the B and D genomes of wheat. None have been observed with the A genome of wheat. The observation that *Am. muticum* preferentially pairs with B and D genome chromosomes indicates that the T genome of the wild relative is more closely related to the progenitors of the B (thought to be *Ae. speltoides* or a close relative – Dvorak and Zhang, 1990) and D genomes (*Ae. tauschii* – McFadden and Sears, 1946) than to the A genome donor (*T. urartu* – Dvorak *et al*., 1993; King *et al*., 1994). A further indicator of a potentially close relationship between *Am. muticum* and *Ae. speltoides* is the fact that both species carry an inhibitor of the *Ph1* locus (Bennett *et al*., 1974; Chen *et al*., 1994; Dover and Riley, 1972; Dvorak, 1972) a phenomenon which is extremely rare. The present SNP analysis does not reveal which wheat chromosomes have been involved in introgressions with *Am. muticum*. Thus, it is presently not possible to determine whether introgressions between the A genome of wheat and the T genome of *Am. muticum* have occurred other than by GISH. However, a future aim of the programme is to produce lines that are homozygous for each of the introgressions generated. Once generated, wheat chromosome‐specific markers will be used to determine which wheat chromosome(s) is (are) involved in each of the introgressions.

In the work described, homoeologous recombination was induced by the *Ph1* suppressor action of *Am. muticum* genes. Previous work postulated that the suppression of *Ph1* pairing control by *Am. muticum* involved two gene loci with two different allelic variants (Bennett *et al*., 1974; Dover and Riley, 1972). In this work, the *Ph1* suppressors resulted in a high frequency of homoeologous recombination during gametogenesis in the F_1_ hybrids. However, homoeologous recombination between the chromosomes of *Am. muticum* and those of wheat was also observed in later generations. We are presently trying to determine the genetic control of the *Am. muticum Ph1* suppression system. This will provide information on whether the genes that suppress the *Ph1* locus were present in lines that underwent further recombination in the BC_1_, BC_2_ and BC_3_ generations.

The wild relatives of wheat provide a vast reservoir of genetic variation for agronomically important traits such as plant production (e.g. photosynthetic capacity), tolerance to abiotic stresses (e.g. heat, drought and salinity) and biotic stresses (e.g. fungal diseases and insect attack). With the lack of diversity within wheat itself, wild relatives may prove to be the primary means by which to increase wheat yields above the current plateau. In this work, we have shown that for the first time, we can systematically unlock the genomewide reservoir of genetic variation available in a wild relative for utilization in wheat breeding. However, for the full value of the material to be recognized, it is essential that the germplasm generated is fully phenotypically characterized.

## Experimental procedures

### Plant material

In order to generate introgressions, hexaploid wheat (variety, Paragon) was pollinated with *Am. muticum* (accessions 2130004, 2130008, 2130012 obtained from JIC stock centre) to produce F_1_ interspecific hybrids (Figure 1). Introgression of genetic variation from *Am. muticum* into wheat occurs when the chromosomes of the two species recombine during gametogenesis in these interspecific F_1_ hybrids. This results in the production of gametes which carry *Am. muticum*/wheat recombinant chromosomes (the subsequent transmission of these recombinant chromosomes to their progeny leads to the generation of *Am. muticum*/wheat introgressions).

The hybrids were then grown to maturity and backcrossed as the female with the wheat parent to generate BC_1_ populations. The BC_1_ individuals and their resulting progenies were then recurrently pollinated with the wheat parent to produce BC_2_, BC_3_ populations, etc. (Figure 1).

### Identification of introgressions via an Affymetrix SNP array

The Nottingham/BBSRC Wheat Research Centre (WRC) is presently engaged in the genomewide introgression of genetic variation from ten wild relatives into wheat, that is *Am. muticum, Ae. speltoides, Aegilops caudata, Triticum timopheevii, T. urartu, Secale cereale, Thinopyrum bessarabicum, Thinopyrum elongatum, Thinopyrum intermedium* and *Thinopyrum ponticum*. To detect introgressed chromosomes and chromosome segments from these wild relatives into wheat, an array of circa 35K SNPs has been developed. In summary, the array is composed of SNPs each showing polymorphism for the ten wild relatives relative to the wheat genotypes understudy. [All the SNPs incorporated in this array formed part of the Axiom^®^ 820K SNP array (Winfield *et al*., 2015). The data set for the Axiom^®^ 820K array is available from www.cerealsdb.uk.net (Wilkinson *et al*., 2012)]. Table 1 shows the number of putative SNPs between *Am. muticum* and each of the wheat genotypes included on the array. The array has been constructed in such a way that up to 384 lines can be screened at one time. Thus, the array facilitates the high‐throughput, high‐resolution screening of introgressions that are being generated from any of the ten wild relatives, including *Am. muticum*.

### Genotyping

The Axiom^®^ Wheat‐Relative Genotyping Array was used to genotype 167 samples using the Affymetrix GeneTitan^®^ system according to the procedure described by Affymetrix (Axiom^®^ 2.0 Assay Manual Workflow User Guide Rev3). Allele calling was carried out using the Affymetrix proprietary software packages Affymetrix Power Tools (APT) and SNPolisher^™^ (http://www.affymetrix.com/estore/partners_programs/programs/developer/tools/devnettools.affx). A custom software pipeline ADAP (Axiom^®^ Data Analysis Pipeline) was written in perl to simplify the data analysis, following the Axiom^®^ Best Practices Genotyping Workflow (http://media.affymetrix.com/support/downloads/manuals/axiom_genotyping_solution_analysis_guide.pdf). A variant call rate threshold of 80% was used instead of the default value (97%) to account for the lower call rates typically obtained from hybridising wheat relatives and progenitors to the array. The apt‐probeset‐genotype program within Affymetrix Power Tools determines genotype calls from Affymetrix SNP microarrays. Following this, the SNPolisher R package calculates SNP performance metrics, such as call rate, cluster separation and deviation from expected cluster position. It then classifies the SNPs into performance categories. These categories were as follows: (i) ‘Poly High Resolution’ (PHR), which were codominant and polymorphic, with at least two examples of the minor allele; (ii) ‘No Minor Homozygote’ (NMH), which were polymorphic and dominant, with two clusters observed; (iii) ‘Off‐Target Variant’ (OTV), which had four clusters, one representing a null allele; (iv) ‘Mono High Resolution’ (MHR), which were monomorphic; (v) ‘Call Rate Below Threshold’ (CRBT), where SNP call rate was below threshold but other cluster properties were above threshold; and (vi)’ Other’, where one or more cluster properties were below threshold. For genetic mapping purposes, only the PHR SNPs were used as they provide good cluster resolution where each SNP essentially behaves like a diploid.

### Genetic Mapping of *Am. muticum* chromosomes

Individuals from a backcross population between *T. aestivum* and *Am. muticum* were genotyped with the Axiom^®^ Wheat‐Relative Genotyping Array. Along with triplicates of the three parental lines, 158 lines comprising BC_1_, BC_2_ and BC_3_ populations of *Am. muticum* were genotyped altogether. As stated above, only the PHR SNP markers were used for genetic mapping. SNP markers which showed (i) heterozygous calls for either parent(s), (ii) no polymorphism between the wheat parents and *Am. muticum* and/or (iii) no calls for either parent(s) were removed using Flapjack^™^ (Milne *et al*., 2010; v.1.14.09.24). The resulting markers were sorted into linkage groups in JoinMap^®^ 4.1 (van Ooijen, 2011) with a LOD score of 20 and a recombination frequency threshold of 0.1 using the Haldane mapping function (Haldane, 1919). All markers that did not show any heterozygous call or were unlinked were ignored and only the highest ranking linkage groups with more than 30 markers were selected for map construction. These were exported and assigned to chromosomes using information from the Axiom^®^ Wheat HD Genotyping Array (Winfield *et al*., 2015). Where chromosomes were split into multiple linkage groups, these were re‐formed into a single linkage group and reordered. Erroneous markers that had more than 20% missing data or showed a unique pattern of segregation that was either not observed in the previous backcross generation or not consistent with the recombination of neighbouring markers in the group, in different samples, were also removed. The long and short arm of each chromosome was identified from the IWGSC wheat survey sequence (The International Wheat Genome Sequencing Consortium, 2014), and groups were orientated to have the short arm above the long arm. Final map reordering was conducted with JoinMap 4.1 and genetic maps produced through MapChart 2.3 (Voorrips, 2002). In some cases, physical map information was employed to order loci. Graphical genotype visualization was performed using Graphical GenoTypes 2.0 (GGT; van Berloo, 2008).

### Comparative analysis

Synteny analysis was carried out using sequence information of the markers located on the present map of *Am. muticum*. The sequences of the mapped markers were compared using BLAST (e‐value cut‐off of 1e‐05) against the wheat genome (http://plants.ensembl.org/Triticum_aestivum) to obtain the orthologous map positions of the top hits in the A, B and D genomes of wheat. To generate the figures, cM distances on the linkage groups of the present map of *Am. muticum* were scaled up by a factor of 100 000 to match similar base pair lengths of the chromosomes of the wheat genome. Figure 6 was visualized using Circos (v. 0.67; Krzywinski *et al*., 2009) to observe synteny between *Am. muticum* (genetic position in cM) and the wheat genome (physical position in Mb).

### Cytogenetic analysis

The protocol for genomic *in situ* hybridisation (GISH) was as described in Zhang *et al*. (2013) and Kato *et al*. (2004). Genomic DNA was isolated using a CTAB method (Zhang *et al*., 2013) from young leaves of the three putative diploid progenitors of bread wheat, that is *T. urartu* (A genome), *Ae. spltoides* (B genome) and *Ae. tauschii* (D genome), and from *Am. muticum*. The genomic DNA of *Am. muticum* and *T. urartu* was labelled by nick translation with Chroma Tide Alexa Fluor 488‐5‐dUTP (Invitrogen; C11397). Genomic DNA of *Ae. tauschii* was labelled with Alexa Fluor 594‐5‐dUTP (Invitrogen; C11400). Genomic DNAs of *Ae. speltoides* and *T. aestivum* cv. Chinese Spring were fragmented to 300–500 bp in boiling water.

Preparation of chromosome spread was as described in Kato *et al*. (2004), with modifications. Roots from each germinated introgression line were excised and treated with nitrous oxide gas at 10 bar for 2 h. Treated roots were fixed in 90% acetic acid for 10 min and then washed three times in water on ice. The root tip was dissected and digested in 20 μL of 1% pectolyase Y23 and 2% cellulase Onozuka R‐10 (Yakult Pharmaceutical, Tokyo) solution for 50 min at 37 °C and then washed three times in 70% ethanol. The root tips were crushed in 70% ethanol, and the cells collected by centrifugation at 3000 × g for 1 min, briefly dried and then re‐suspended in 30–40 μL of 100% acetic acid before being placed on ice. The cell suspension was dropped onto glass slides (6–7 μL per slide) in a moist box and dried slowly under cover.

Slides were initially probed using labelled genomic DNA of *Am. muticum* 10(0 ng) and fragmented genomic DNA of Chinese Spring (3000 ng) as blocker to detect the *Am. muticum* introgressions. Probe to block was in a ratio of 1–30 (the hybridization solution was made up to 10 μL with 2 × SSC in 1 × TE). The slides were then bleached and re‐probed with labelled DNAs of *T. urartu* (100 ng) and *Ae. taushii* (200 ng) and fragmented DNA of *Ae. speltoides* (5000 ng) as blocker in the ratio 1–2 to 50 to detect the AABBDD genomes of wheat. All slides were counterstained with DAPI and analysed using a Leica DM5500B epifluorescence microscope (Leica Microsystems, Wetzlar, Germany) with filters for DAPI (blue), Alexa Fluor 488 (green) and Alexa Fluor 594 (red). Photographs were taken using a Leica DFC 350FX digital camera.

## Supporting information


**Table S1.** Number of seed produced and germinated in relation to number of crosses carried out for each generation of the introgression program for *Am. muticum* into wheat.

**Table S2**. SNP marker names and cM position on the genetic linkage map of *Am. muticum* shown in Fig. 2.
