# Supplementary material for: A step change in the transfer of interspecific variation into wheat from Amblyopyrum muticum
Source: Plant Biotechnol J. 2016 Aug 25;15(2):217–26. doi: 10.1111/pbi.12606 (PMC5258861; doi:10.1111/pbi.12606)
Supplement: Supplementary file 1 — Table S1. Number of seed produced and germinated in relation to number of crosses carried out for each generation of the introgression program for Am. muticum into wheat. [file PBI-15-217-s002.docx]

**Table S1.** Number of seed produced and germinated in relation to number of crosses carried out for each generation of the introgression program for *Am. muticum* into wheat.

|  | Paragon x *Amblyopyrum muticum* | F_1_ | BC_1_ | BC_2_ | BC_3_ | Totals |
| --- | --- | --- | --- | --- | --- | --- |
| Number of seed sown | NA | 98 | 34 | 116 | 127 | 375 |
| Number of seed that germinated  (%) | NA | 28  (28.6) | 18  (52.9) | 90  (77.59) | 77  (60.63) | 213 |
| Number of plants setting seed  (%) | NA | 11  (39.29) | 16  (88.89) | 87  (96.67) | 64  (83.12) | 178 |
| Number of seed/total number of crosses  (Average number of seed set per crossed ear) | 211/64  (3.3) | 34/136  (0.25) | 781/123  (6.35) | 4173/487  (8.57) | 2947/229  (12.9) | 8146/1039 |
| Number of crosses producing seed  (%) | 36  (56.25) | 22  (16.18) | 98  (79.67) | 429  (88.09) | 225  (98.25) | 810 |
| Number of self seed produced | 0 | 0 | 76 | 1816 | 2085 | 3977 |
